# Neuromuscular deterioration in the early stage of sepsis in rats

**DOI:** 10.1186/cc5139

**Published:** 2007-01-04

**Authors:** Ilkin Cankayali, Yusuf Hakan Dogan, Ilhami Solak, Kubilay Demirag, Oguz Eris, Serdar Demirgoren, Ali Resat Moral

**Affiliations:** 1 Department of Anaesthesiology and Intensive Care Unit, Ege University, School of Medicine 35100, Izmir, Turkey; 2 Department of Physiology, Ege University, School of Medicine 35100, Izmir, Turkey; 3 Department of General Surgery, Ege University, School of Medicine 35100, Izmir, Turkey

## Abstract

**Introduction:**

Critical illness polyneuropathy (CIP) is a clinical condition frequently seen in patients being treated in critical care units in the final stage of sepsis. The etiopathology of CIP is still unclear, and the onset time of appearance of the electrophysiological findings has not been elucidated. The very little research that has been carried out on this topic is limited to clinical electrophysiological and histopathological studies. In this study, electrophysiological alterations in the early stage of experimentally induced sepsis were investigated in septic rats.

**Methods:**

We conducted a prospective, randomized, controlled experimental study in an animal basic science laboratory with 30 male Sprague-Dawley rats, weighing 200 to 250 g. All of the rats were randomly assigned to one of two groups. In the sepsis group (*n *= 20), cecal ligation and puncture (CLP) was performed to induce experimental sepsis. In the sham-operated group (*n *= 10), laparotomy without CLP was performed. Before and 24 hours after CLP and laparotomy, the right sciatic nerve was stimulated from the sciatic notch and compound muscle action potentials (CMAPs) were recorded from the gastrocnemius muscle. Recordings of latency, amplitude, and duration of the CMAP were evaluated.

**Results:**

CMAP durations before and 24 hours after surgery were 0.45 ± 0.05 ms and 0.48 ± 0.05 ms, respectively, in the sham-operated group and 0.46 ± 0.05 ms and 0.55 ± 0.01 ms, respectively, in the sepsis group. Latency measurements in the sham-operated group were 0.078 ± 0.010 ms and 0.080 ± 0.015 ms, respectively, whereas measurements were found to be prolonged in the sepsis group: 0.094 ± 0.015 ms and 0.149 ± 0.054 ms before and 24 hours after surgery, respectively (*p *< 0.05). CMAP amplitudes in the sham-operated group before and 24 hours after surgery were 8.41 ± 0.79 mV and 8.28 ± 1.92 mV, respectively, whereas in the sepsis group the amplitude measurements decreased to 7.60 ± 1.75 mV and 4.87 ± 3.44 mV, respectively (*p *< 0.05).

**Conclusion:**

The results of the study indicate that electrophysiological alterations appear in the first 24 hours after experimental sepsis and are characterized by an increase in latency and a decrease in CMAP amplitude. The results also suggest that electrophysiological findings seen in patients with CIP might appear before clinical signs of CIP.

## Introduction

Critical illness polyneuropathy (CIP) was described as a clinical disorder by Bolton and colleagues [1] in 1984. It is a primary axonal degeneration of motor and sensory fibers which occurs mostly in patients who have systemic inflammatory response syndrome (SIRS), sepsis, or multiple organ dysfunction syndrome (MODS) [2-7]. Berek and colleagues [8] suggested that, in the course of sepsis, CIP has to be considered part of MODS.

CIP manifests with general weakness and sensory defects and especially with weakness of the respiratory muscles, leading to problems in weaning from mechanical ventilation in the intensive care unit (ICU). Physiopathology, onset of the symptoms, and the treatment of CIP have not been clearly defined.

The studies on CIP are based mostly on clinical manifestations and neurophysiological research. However, an experimental study investigating possible neuromuscular changes in the early stage of sepsis has not been performed yet. We aimed to observe electrophysiological alterations in the early stage of sepsis. In this study, an experimental sepsis model was performed to investigate electrophysiological alterations in the first 24 hours of sepsis.

## Materials and methods

Animal Ethics Committee approval was obtained, and the study was conducted in the Research Laboratory of the Department of Anesthesiology and ICU of Ege University Medical School (Izmir, Turkey).

### Experimental procedures

Thirty adult male Sprague-Dawley rats two to three months old, each weighing approximately 250 g, were used. All rats were housed in cages one week before the experiments in an acclimatized room at standard room temperature and with twelve hour light/dark cycles. Rats were allowed free access to water and standard chow. For surgical intervention, rats were anesthetized with ketamine (80 mg/kg) and xylazine (10 mg/kg) given intraperitoneally.

All rats were randomly divided into one of two groups: a cecal ligation and puncture (CLP)-operated group (sepsis group) (*n *= 20) and a sham-operated group (sham group) (*n *= 10). Due to the sepsis model's high mortality rate, more rats were grouped in the sepsis group (*n *= 20) than in the sham group (*n *= 10). For reliable statistical results, at least six rats is sufficient. We decided to perform the study with at least 10 rats.

Sepsis was induced by CLP performed as described previously [9,10]. In this sepsis model, five hours after CLP, rats were accepted as septic. Under aseptic conditions, a 3-cm midline laparotomy was performed to allow exposure of the cecum with adjoining intestine. The cecum was ligated tightly with a 3.0 silk suture at its base below the iliocecal valve and perforated once with a 22-gauge needle. The cecum was then gently squeezed to extrude a small amount of feces from the perforation site. The cecum was returned to the peritoneal cavity, and the laparotomy incision was closed with 4.0 silk sutures. In the sham group, under aseptic conditions only, laparotomy was performed on rats, but their cecum was neither ligated nor punctured.

### Measurements and calculations

**Figure 1:**
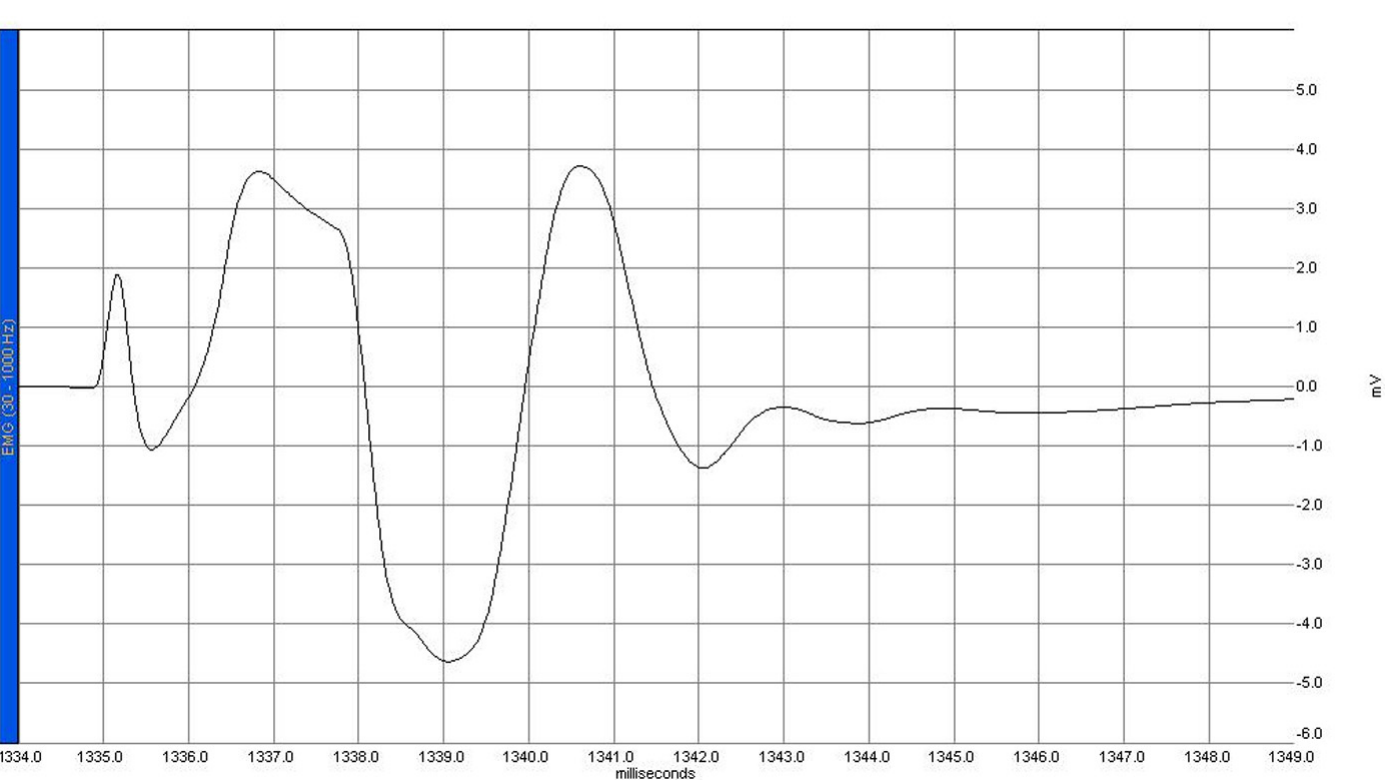
A sample of compound muscle action potential recorded before laparotomy in the sham group. EMG, electromyelograph.

**Figure 2:**
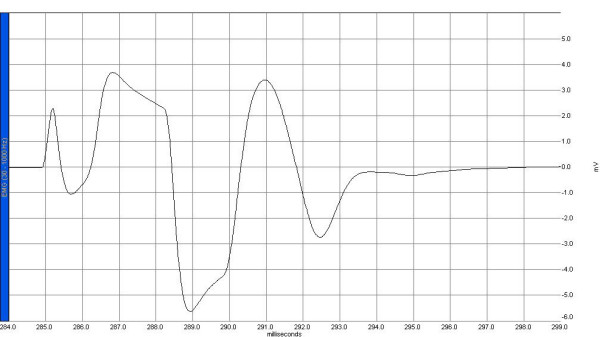
A sample of compound muscle action potential recorded 24 hours after laparotomy in the sham group. EMG, electromyelograph.

**Figure 3:**
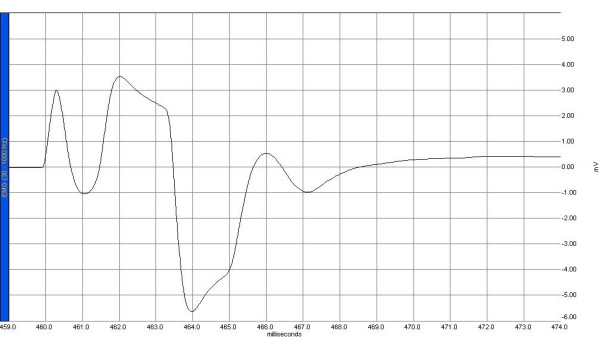
A sample of compound muscle action potential recorded before cecal ligation and puncture in the sepsis group. EMG, electromyelograph.

**Figure 4:**
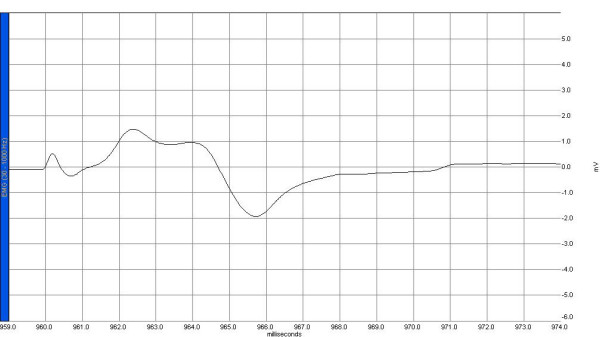
A sample of compound muscle action potential sample recorded 24 hours after cecal ligation and puncture in the sepsis group. EMG, electromyelograph.

Electrophysiological recordings were obtained from the right sciatic nerve stimulated supra-maximally (intensity 10 V, duration 0.1 ms, frequency 1 Hz) by a Biopac HSTM01 surface stimulation electrode (BIOPAC Systems, Inc., Santa Barbara, CA, USA) from the sciatic notch, and compound muscle action potentials (CMAPs) were recorded by means of superficial disc electrodes located over the gastrocnemius muscle before and 24 hours after surgery. Data were evaluated using Biopac Student Lab Pro version 3.6.7 software (BIOPAC Systems, Inc.), with latency, amplitude, and duration of CMAP as the parameters (Figures 1, 2, 3, 4). During the electromyelographic (EMG) recordings, rectal temperatures of the rats were monitored by a rectal probe (HP Viridia 24-C; Hewlett-Packard Company, Palo Alto, CA, USA) and the temperature of each rat was kept at approximately 36°C to 37°C by heating pad. The animals were euthanized 24 hours after the CLP for the next recording.

Because we aimed to assess EMG recordings in the early stage of sepsis, we obtained EMG recordings of the rats in the first 24 hours after CLP. We did not aim to observe clinical signs of sepsis; therefore, the animals were euthanized 24 hours after surgery.

### Statistical analysis

The results were analyzed with the SPSS ver 14.0 statistical program (SPSS Inc., 233 South wacker Drive, 11^th ^floor, Chicago, IL 60606-6307) by using repeated measures (analysis of variance). Factors were session (before and 24 hours after surgery) and treatment (sepsis and sham groups). Dependent variables were latency, amplitude, and duration. The groups were compared by paired-sample *t *test, and results were given as mean ± standard deviation. A value of *p *> 0.05 was accepted as statistically significant.

## Results

In the sepsis group, five rats died during the first 24 hours and were excluded from the study. At 24 hours, the mortality rate was 25% in the sepsis group, and there was no mortality in the sham group. The mortality rate was high in the sepsis group because we did not use treatment materials (antibiotics and fluid resuscitation) for this study.

CMAP durations before and 24 hours after surgery were recorded as 0.45 ± 0.05 ms and 0.48 ± 0.05 ms, respectively, in the sham group. Statistically significant difference was not found (*p *> 0.05). CMAP durations before and 24 hours after surgery were recorded as 0.46 ± 0.05 ms and 0.55 ± 0.01 ms, respectively, in the sepsis group. Statistically significant difference was found in CMAP duration only for session (F_1,23 _= 7.49, *p *= 0.012) but not for treatment (F_1,23 _= 4.02, *p *= 0.057) (*p *> 0.05) (Table 1). CMAP amplitudes in the sham group before and 24 hours after surgery were 8.41 ± 0.79 mV and 8.28 ± 1.92 mV, respectively. Statistically significant difference was not found (*p *> 0.05) (Table 1). However, in the sepsis group, the amplitudes decreased from 7.60 ± 1.75 mV to 4.87 ± 3.44 mV. This alteration was statistically significant (*p *< 0.05) (Table 1). CMAP amplitudes in the sham group were not different statistically but session (F_1,23 _= 5.56, *p *= 0.027) and treatment (F_1,23 _= 8.40, *p *= 0.008) were significantly interacted (F_1,23 _= 4.38, *p *= 0.047) and the effect was observed only in the sepsis group (Table 1). Whereas CMAP amplitudes decreased profoundly in the sepsis group (ratio of the prolonged time = -33.3%), CMAP amplitudes were much less decreased in the sham group (ratio of the prolonged time = -0.7%).

**Table 1:** Measurements of compound gastrocnemius muscle action potentials

	CMAP duration (ms)		CMAP amplitude (mV)		Latency (ms)	
Groups	Before surgery	24 hours after surgery	Before surgery	24 hours after surgery	Before surgery	24 hours after surgery

Sepsis group	0.46 ± 0.05	0.55 ± 0.01	7.60 ± 1.75	4.87 ± 3.44^a^	0.094 ± 0.015	0.149 ± 0.054^a^
Sham group	0.45 ± 0.005	0.48 ± 0.05	8.41 ± 0.79	8.28 ± 1.92	0.078 ± 0.010	0.080 ± 0.015

^a^*p *< 0.05. All values are presented as mean ± standard deviation for each group. CMAP, compound muscle action potential.

Latency measurements were not significantly altered in the sham group (from 0.078 ± 0.010 ms to 0.080 ± 0.015 ms), whereas measurements were found to be prolonged in the sepsis group (from 0.094 ± 0.015 ms to 0.149 ± 0.054 ms) before and 24 hours after surgery, respectively (*p *< 0.05). Latency data showed significant difference only in the sepsis group, and interacted for session (F_1,23 _= 13.47, *p *= 0.001) and treatment (F_1,23 _= 15.86, *p *= 0.001) (F_1,23 _= 11.98, *p *= 0.002). Whereas latency time was prolonged in the sepsis group in a significant manner (ratio of the prolonged amount = 56.45%), latency time was prolonged much less in the sham group (ratio of the prolonged amount = 2.4%) (Table 1).

CMAP duration and latency increase and CMAP amplitude decrease in the sham group was not statistically significant (*p *> 0.05).

## Discussion

CIP is a neuromuscular pathology regarded as a neurological complication of sepsis given that CIP may have always accompanied sepsis [3-6,8,11,12]. Underlying primary illness or the type of trauma, metabolic disorders, hypoxia, nutritional deficiencies, and medications such as antibiotics, neuromuscular blocking agents, and corticosteroids are insufficient to reveal the potential causes of CIP [2,4]. However, etiology, pathogenesis, time of onset, preventive measures, and therapy of CIP are still controversial and could not be defined clearly. In patients with sepsis and SIRS, the seriousness of the underlying disease and treatments with neuromuscular drugs and opioids and mechanical ventilation may conceal the onset and symptoms of CIP and may delay the diagnosis of CIP. Therefore, electrophysiological examination is the most important tool in the early diagnosis and course of CIP [2,13]. But the muscle fibrillation potentials and positive sharp waves cannot be observed before three weeks of sepsis. Otherwise, the latency changes that are accepted as typical for axonal damage and the decrease of the motor action potential (CMAP) amplitude may appear in the first week of sepsis. They are regarded as the earliest electrophysiological signs of CIP. Despite denervation, signs have been found on the fifth day; in some studies, spontaneous EMG activity cannot be expected before the 10^th ^or 14^th ^day of acute denervation [14]. In the electrophysiological research of Tennila and colleagues [15], CMAP amplitudes of median and ulnar nerves were found to be decreased on the fifth day in nine mechanically ventilated patients with SIRS and/or MODS. In addition, abnormal spontaneous activities such as sharp positive waves and fibrillation potentials were found to be present in EMG recordings in their clinical series.

In this study, CMAP amplitude was decreased; regarding the first electrophysiological finding in the early phase of CIP [2,6,16] and marked prolongation of latency values appeared in the first 24 hours of sepsis. Our findings are supported by the previous studies. In some earlier studies, it was postulated that decrease in the CMAP amplitude was due primarily to neuromuscular blocking agents, steroids, and some medications used widely in ICUs [4,17-19]. However, the latest prospective studies have shown that there is no correlation with CIP, critical illness myopathy, and medication used [5,6,20]. In the present study, the neurophysiological conduction alterations were seen in the experimental CIP model without using neuromuscular blocking agents and/or steroids. Therefore, these findings support the concept that sepsis is mainly responsible for the neuromuscular changes. Likewise, antibiotics, especially aminoglycosides and their metabolites, are said to be the other factors responsible for development of CIP that results from the increase of capillary membrane permeability and the invasion of antibiotics to peripheral nerves in sepsis [21]. However, there is no statistical evidence confirming these opinions, and thus further studies are needed. Given that antibiotics were not used in our experimental sepsis model, our results support the idea that the observed electrophysiological alterations were attributable entirely to sepsis.

Disturbances in microcirculation and autoregulation of peripheral nerves, as well as the other organs influenced in sepsis, are thought to be the principal causes of the development of CIP. In addition, cytokines released in sepsis also cause an increase in capillary permeability due to a histamine-like effect and the resulting endoneural edema leads to hypoxia and energy deficit by increasing the intercapillary space [2]. Because the axonal transportation of structural proteins is highly energy-dependent, this energy deficit induces primary axonal degeneration of distal nerves [2]. Bolton and colleagues [3] suggested that tumor necrosis factor, arachidonic acid, and metabolites of histamine, complement activation, cellular adhesion systems, and free radicals were principal factors responsible for systemic effects of sepsis and SIRS and these factors might lead to primary axonal degeneration.

Electrophysiological measurements in the early studies obtained during early periods of clinical sepsis indicated that the decrease in amplitude of CMAP was accompanied by an increase in duration without any change in latency. This finding directed attention to the muscle fiber membrane as a physiopathological explanation [22]. Decrease in CMAP amplitude and increase in duration were suggested to be secondary to the dysfunction of energy-dependent sodium-potassium pumps in muscles [23]. In the present study, although there was an increase in the duration 24 hours after sepsis was induced, the difference was not statistically significant when compared with the sham group. Our results do not show the prolongation of duration which has been shown in the previous studies, due to electrophysiological data that were obtained in the early stages (24 hours) of sepsis in this study. The prolongation of the duration due to sodium-potassium pump insufficiency in the muscles has been accepted as an indicator observed in the later stages of sepsis. Our results indicate no change in CMAP duration but a decrease in amplitude and observable prolongation of the latency which is regarded as an indicator of axonal degeneration. Further detailed studies should be designed to elucidate the pathogenesis properly. The common point in the majority of the related articles is the presence of a decrease of CMAP amplitude. CMAP is produced by synchronized activation of the muscle fibers after axonal innervations, which is the sum of the responses of the striated muscles to stimuli. In addition, CMAP is a valuable tool both for evaluating to descending motor axon and the response of the muscle fibers to the stimulus placed distally and the conduction at the neuromuscular junction [24]. In this study, CMAP changes indicate possible axonal conduction and/or neuromuscular junction pathologies or a reduced number of fibers responding to stimulus. But we were not able to distinguish and define the origin of the observed changes such as axonal conduction defects, neuromuscular junction pathologies, or reduction of the number of the muscle fibers that led to the decrease of the amplitude and prolongation of CMAP in the sepsis group.

## Conclusion

Our results indicate that electrophysiological findings appeared in the first 24 hours after experimental sepsis and were characterized by an increase in latency and a decrease in CMAP amplitude. Therefore, we conclude that electrophysiological changes seen in sepsis might appear before clinical signs of CIP.

## Key messages

• CMAP durations were increased in a sepsis model in rats in the first 24 hours.

• CMAP amplitudes were significantly decreased in a sepsis model in rats in the first 24 hours.

• Latency times were significantly prolonged in a sepsis model in rats in the first 24 hours.

• Electrophysiological changes seen in sepsis might appear before clinical signs of CIP.

## Abbreviations

CIP = critical illness polyneuropathy; CLP = cecal ligation and puncture; CMAP = compound muscle action potential; EMG = electromyelographic; ICU = intensive care unit; MODS = multiple organ dysfunction syndrome; SIRS = systemic inflammatory response syndrome.

## Competing interests

The authors declare that they have no competing interests.

## Authors' contributions

IC, IS, and ARM designed the study. IC, YHD, IS, KD, OE, and ARM coordinated the study and drafted the manuscript. IC, YHD, and IS collected data. IC, IS, YHD, KD, OE, SD, and ARM helped to draft the manuscript. IC, YHD, IS, and ARM conceived and designed the study and performed the statistical analysis. All authors read and approved the final manuscript.
